# SARS-CoV-2 anti-RBD and anti-N protein responses are differentially regulated between mother-child pairs: insight from a national study cohort at the Faroe Islands

**DOI:** 10.3389/fimmu.2024.1418678

**Published:** 2024-07-03

**Authors:** Ida Jarlhelt, Cecilie Bo Hansen, Laura Pérez-Alós, Pál Weihe, Maria Skaalum Petersen, Peter Garred

**Affiliations:** ^1^ Department of Research, The National Hospital of the Faroe Islands, Tórshavn, Faroe Islands; ^2^ Center of Health Science, University of the Faroe Islands, Tórshavn, Faroe Islands; ^3^ Laboratory of Molecular Medicine, Department of Clinical Immunology, Section 7631, Copenhagen University Hospital, Rigshospitalet, Copenhagen, Denmark; ^4^ Department of Clinical Medicine, Faculty of Health and Medical Sciences, University of Copenhagen, Copenhagen, Denmark

**Keywords:** SARS-CoV-2, antibody duration, maternal immunization, nucleocapsid protein, spike protein

## Abstract

**Background:**

Knowledge about SARS-CoV-2 antibody dynamics in neonates and direct comparisons with maternal antibody responses are not well established. This study aimed to characterize and directly compare the maternal and infant antibody response in a national birth cohort from the Faroe Islands.

**Methods:**

The levels of immunoglobulins (Ig) targeting the receptor binding domain (RBD) of the spike protein and the nucleocapsid protein (N protein) of SARS-CoV-2 were investigated in maternal blood and umbilical cord blood from neonates. The study included 537 neonates and 565 mothers from the Faroe Islands, and follow-up samples were collected 12 months after birth. Multiple linear regression models were used to assess associations of maternal parameters with maternal and neonatal Ig levels and pregnancy outcomes.

**Results:**

The finding showed that neonates acquired varying levels of SARS-CoV-2 antibodies through transplacental transfer, and the levels were significantly influenced by the mother’s vaccination and infection status. The study also found that maternal vaccination and the presence of SARS-CoV-2 antibodies targeting spike RBD were associated with gestational age and APGAR scores. Furthermore, the anti-RBD and -N protein-specific antibody response dynamics during 12 months after birth exhibited differences between mothers and children. RBD and N protein responses were maintained at follow-up in the mother’s cohort, while only the N protein response was maintained at follow-up in the children’s cohort.

**Conclusion:**

In conclusion, SARS-CoV-2-specific immune responses in newborns rely on maternal immunity, while the persistence of SARS-CoV-2-specific Igs appears to be differently regulated between mothers and children. The study provides new insights into the dynamics of SARS-CoV-2-specific immune responses in newborns and underscores the nuanced relationship between maternal factors and neonatal humoral responses.

## Introduction

Coronavirus disease 2019 (COVID-19) is caused by severe acute respiratory syndrome coronavirus 2 (SARS-CoV-2), which has spread across every continent since the initial outbreak at the end of 2019, with more than 700 million confirmed cases by the time of manuscript preparation (1).

The global impact of the SARS-CoV-2 virus has encouraged extensive research to unravel its various facets, including the intricate dynamics of maternal and neonatal immune responses. The viral antibody response differs between infants and adults, owing to several factors, including the developing infant immune system and the absence of immunological memory (2). Infant-specific antibody responses to SARS-CoV-2 have been less explored than other areas of COVID-19. An important aspect of immunity against infectious pathogens in infants relies on effective maternal antibody production and their transfer across the placenta to provide passive immunity in the infant. Several studies have demonstrated the transplacental transfer of maternal SARS-CoV-2 antibodies (3–5). In addition, prenatal COVID-19 vaccination is recommended, and it is now widely accepted that vaccination before and during pregnancy generates functional IgG in the maternal circulation that can be detected in umbilical cord blood at birth, protecting neonates from severe COVID-19 infection (6–8). A recent multi-omics analysis of mucosal and systemic immunity to SARS-CoV-2 after birth showed that infection stimulated robust antibody titers that, unlike in adults, showed no sign of decay for up to 300 days (9). Another study investigating the risk of SARS-CoV-2 infection in children with mothers vaccinated during pregnancy found that passively transferred maternal IgG was protective against infant SARS-CoV-2 infection, with higher antibody levels at birth significantly associated with longer disease-free intervals (10). Therefore, understanding the transmission and development of antibodies in both mothers and their newborn children is crucial for elucidating the potential protective mechanisms and vulnerability within this susceptible population.

We aimed to provide a comprehensive analysis of SARS-CoV-2 antibody responses in mothers and their newborns, shedding light on the intricate interplay between maternal immunity and neonatal immune development. This was achieved by examining the levels of SARS-CoV-2 Ig against receptor binding domain (RBD) of the spike protein and nucleocapsid protein (N protein) in maternal blood and infant umbilical cord blood, using a national birth cohort from the Faroe Islands. Distinguishing between vaccine-induced and infection-induced COVID-19 immunity has been challenging due to the shared S protein component in both vaccines and the virus itself. All approved vaccines in Europe and the USA use the viral S protein, which is also commonly targeted in immunoassays for SARS-CoV-2 antibodies. Instead, detecting antibodies against the N protein can indicate past infection and is unaffected by vaccination status. Using an N protein-specific assay alongside an RBD-specific assay enhances the accuracy of SARS-CoV-2 immunity assessments, allowing differentiation between natural infection and vaccine-induced seropositivity.

The study integrates clinical, serological, and epidemiological data to examine the nuanced aspects of SARS-CoV-2 antibody responses, hopefully contributing valuable insights that can inform public health interventions and enhance our overall understanding of the virus’s impact on this specific demographic.

## Materials and methods

### Study population and sample collection

The study was based on a population-based prospective cohort from the Faroe Islands, the Faroese Birth Cohort 6. The Faroese Cohort includes 629 full-term children-mother pairs, representing 61% of all invited. Participants were consecutively recruited at the National Hospital in Tórshavn, Faroe Islands, between September 2020 and September 2022. Due to the limited amount of samples available, some individuals were excluded from the study. The resulting cohort included 537 children with umbilical cord blood collected immediately after delivery and 565 paired mothers with a blood sample collected at week 32 of pregnancy. An electronic self-report questionnaire included questions regarding the course of pregnancy, medical history, and current health. Obstetric information, including maternal age and body mass index (BMI), together with neonatal parameters such as sex, gestational weeks, birth weight, and vaccination data, was extracted from medical records. All mothers were confirmed positive for SARS-CoV-2 using reverse transcription polymerase chain reaction (RT-PCR), with data provided by the Chief Medical Officer’s Office. The blood samples collected for women at 32 weeks of pregnancy and for children after birth are referred to as baseline samples. The cohort children and mothers were invited for a follow-up visit after 12 months. The follow-up included a clinical examination, including measurements of the height and weight of the children, in addition to venous blood sampling from consenting mothers and paired children. The mothers completed an electronic self-report questionnaire, including questions about breastfeeding. Our follow-up study included blood samples from 138 children and 157 mothers collected at 12-month follow-up that were seropositive at baseline. All women provided informed written consent at each visit. The Faroese Research Ethical Committee approved the study, which was performed according to the Faroese Data Protection Legislation. **Supplementary Figure S1A** includes an overview of the study.

### Buffers

The following buffers were used: PBS (10.1 mM Na_2_HPO_4_, 1.5 mM KH_2_PO_4_, 137 mM NaCl, 2.7 mM KCl), PBS-T (PBS, 0.05% Tween 20 [8221840050, Merck]), and dilution buffer (PBS-T, 5 mM EDTA (EDS-500G, Merck), 5% skim milk (70166, Merck)).

### Anti-N protein Ig sandwich-ELISA

Levels of total Ig against N protein were measured using a validated ELISA-based assay as described elsewhere (11). Briefly, Nunc MaxiSorp flat-bottom 96-well plates (442404; Thermo Fisher Scientific) were coated with 0.5 μg/mL N protein in PBS and incubated overnight at 4°C. Plates were blocked with PBS-T for 30 minutes at room temperature (RT). Clinical samples and control sera were diluted 1:2 in dilution buffer and incubated for 1 hour (h) shaking at RT. A positive control pool consisted of sera from N-positive individuals and a negative control pool of healthy individuals with blood drawn before the SARS-CoV-2 emergence. Ig bound to SARS-CoV-2 N protein was detected using 40 ng/mL biotinylated N protein diluted in PBS-T + 5% bovine serum and incubated for 1h shaking at RT. Pierce HRP-conjugated high-sensitivity streptavidin (21130; Thermo Fisher Scientific) was used for detection, diluted 1:16,000 in PBS-T, and incubated for 1h shaking at RT. TMB One substrate (4380A; Kementec) was applied and allowed to react for 30 min. The reaction was stopped with 0.3 M H_2_SO_4_, and the optical density (OD) was measured at 450 nm using a reference wavelength of 630 nm on a GloMax^®^-Multi Detection System (Promega). Plates were washed three times with 400 µl PBS-T between incubation steps. Due to a change in laboratory facilities and absorbance reader equipment, we reassessed the positivity threshold using the cohort from this study. Specifically, 100 mothers with PCR-confirmed SARS-CoV-2 infection and 100 mothers who were neither infected nor vaccinated were included in this re-evaluation. The determined positivity thresholds for the assays are depicted in **Supplementary Figure S2**.

### Anti-RBD Ig sandwich-ELISA

Levels of total Ig against spike RBD were measured using a validated ELISA-based assay as described elsewhere with minor modifications (12). Briefly, Nunc MaxiSorp flat-bottom 96-well plates (442404; Thermo Fisher Scientific) were coated with 0.5 μg/mL RBD in PBS overnight at 4°C. Plates were blocked with PBS-T for 1 h shaking at RT. Samples diluted 1:2 in PBS-T were applied to plates and incubated for 1 h shaking at RT. Biotinylated RBD was then added in a concentration of 0.5 μg/mL in PBS-T and incubated for 1 h shaking at RT. Anti-SARS-CoV-2 RBD Igs were detected using Pierce HRP-conjugated high-sensitivity streptavidin, diluted 1:16,000 in PBS-T, and incubated for 1 h shaking at RT. TMB One was used as a substrate and allowed to react for 5 min. The reaction was stopped with 0.3 M H_2_SO_4_, and the OD was measured as described above. Plates were washed three times with PBS-T between incubation steps.

### Anti-RBD direct ELISA for detection of IgM, IgG, and IgA

IgM, IgG, and IgA levels against SARS-CoV-2 spike RBD were measured using a validated in-house ELISA-based assay described elsewhere (12). Briefly, Nunc-Maxisorp 384-well plates (464718; Thermo Fisher Scientific) were coated with 1 µg/mL RBD in PBS overnight at 4°C. Plates were blocked with PBS-T for 1 h at RT. Subsequently, diluted serum samples were applied in dilution buffer and incubated for 1 h shaking at RT. Antibodies were detected by adding 0.5 µg/mL HRP-conjugated polyclonal rabbit anti-IgM, IgG, or IgA (P0215, P0214, and P0216, respectively, all from Agilent Technologies) and incubated for 1 h shaking at RT. Plates were developed for 7 min (IgG) or 10 min (IgM, IgA) using TMB-One as a substrate. The reaction was stopped using 0.3 M H_2_SO_4_. A mixture containing equal concentrations of recombinant human IgM, IgG, and IgA antibodies against RBD was used as a calibrator (1:2000 dilution; A02046–100, A02038–100, and A02071–100, respectively, all from Genscript). The OD was measured as described above. Plates were washed three times with PBS-T between incubation steps.

Interpolation of circulating IgM, IgG, and IgA levels was performed using GraphPad Prism version 9.3.1 (GraphPad Software) by utilizing non-linear regression with four-parameter curve fitting. Interpolated levels of IgM, IgG, and IgA antibodies were given in arbitrary units per milliliter (AU/mL), with 200 AU/mL being the antibody level given at the lowest dilution of the calibrator. The assay positivity threshold in AU/mL was established in a subsequent study employing the same assay, with values set at 200, 225, and 100 AU/mL for IgM, IgG, and IgA, respectively (13).

### Variant specificity

The specificity of the RBD-specific antibodies towards different SARS-CoV-2 variants was studied using the direct ELISA for detection of IgG as described above, with minor modifications. Nunc MaxiSorp flat-bottom 96-well plates (442404; Thermo Fisher Scientific) were coated with either ancestral (Wildtype) spike RBD [in-house produced as described elsewhere (12)] or one of two Omicron variants, B.1.1.529 S RBD (17885928, Thermo Fisher Scientific) and BA.2 S RBD (17895928, Thermo Fisher Scientific). Subsequent steps of the assay were performed as described above for the detection of SARS-CoV-2 IgG antibodies.

### Statistical analyses

Statistical analyses were performed using R (version 4.1.0 for Windows, R Foundation for Statistical Computing). Statistical differences between categorical data were performed using the chi-square test. Statistical differences between non-matched, non-normally distributed data were performed using the Mann-Whitney test U test or the Kruskal-Wallis test as appropriate. Statistical differences between matched, non-normally distributed data were performed using the Wilcoxon signed-rank test or the Friedman test as appropriate. Multiple comparison analyses were performed using Dunn’s multiple comparison test. Correlations between maternal and neonatal serum antibodies and correlations between the different variant RBDs were performed using the Spearman Rank test. RBD and N protein-specific IgG, IgM, and IgA levels were log10 transformed. Multiple linear regression analyses were used to analyze the influence of maternal parameters on SARS-CoV-2 antibody levels and pregnancy outcomes. Mother’s age, BMI, RBD, and N protein-specific Ig levels were used as continuous predictor variables, while maternal vaccination status, infection status, comorbidities, and medication intake were used as categorical predictor variables defined as “yes” or “no” input. Pregnancy outcomes included weeks of gestation, birth weight, and APGAR scores used as continuous predictor variables. Delivery was used as a categorical predictor variable defined by “cesarean section” or “vaginal delivery” in a logistic regression model. Most of the analyses were adjusted for sex and BMI. Statistical analyses were performed two-sided and a p-value <0.05 was considered significant.

## Results

### Characteristics of the study population

Demographic data and characteristics of the mothers are depicted in **Table 1**. A simplified overview of key maternal characteristics is depicted in **Supplementary Figure S1B**. The study included 565 women with a baseline blood sample collected at week 32 of pregnancy. The mothers had a median age of 31 (IQR: 28–34) years and a median BMI of 25 (IQR: 22.3–28.5) before pregnancy. At the time of the baseline blood collection, a total of 157 (27.8%) women were vaccinated with at least one dose of the Pfizer/BioNTech vaccine. In comparison, 78 (13.1%) had a positive RT-PCR test at any time before baseline blood collection. Data regarding self-reported comorbidities and intake of medication is provided in **Table 1**. A total of 157 mothers with a positive antibody response against either RBD or N protein at baseline were selected for further analysis of Ig levels after 12 months. At the 12-month follow-up, 146 (93%) women were vaccinated with at least one dose, while 124 (79%) women had a positive RT-PCR test at any time before follow-up blood collection. The mothers reported in the questionnaire that 141 (89.8%) had provided breastmilk for their children after birth. Specifically, 17.8% provided breastmilk for less than 26 weeks, while 72.0% provided it for 26 weeks or more. A total of 10.2% of women did not provide breastmilk to their children. Of all women, 52.2% were still partly breastfeeding at the time of follow-up examination (**Table 1**).

**Table 1:** Demographic data and characteristics of mothers.

Mothers at 32 weeks of pregnancy	Infected		Not infected		Total
	Not vaccinated	Vaccinated	Not vaccinated	Vaccinated	(N=565)
	(N=11)	(N=67)	(N=397)	(N=90)	
Age (years)					
median (IQR)	31.0 (25.5 – 33.0)	30.5 (26.0 – 32.8)	31.0 (28.0 – 34.0)	31.0 (28.0 – 34.0)	31.0 (28.0 – 34.0)
N/A	0 (0%)	1 (1.5%)	23 (5.8%)	2 (2.2%)	26 (4.6%)
Age (years)					
<28	4 (36.4%)	23 (34.3%)	86 (21.7%)	21 (23.3%)	134 (23.7%)
>28–34	4 (36.4%)	28 (41.8%)	176 (44.3%)	43 (47.8%)	251 (44.4%)
>34	3 (27.3%)	15 (22.4%)	112 (28.2%)	24 (26.7%)	154 (27.3%)
N/A	0 (0%)	1 (1.5%)	23 (5.8%)	2 (2.2%)	26 (4.6%)
BMI					
median (IQR)	24.2 (22.9 – 25.2)	24.3 (21.8 – 28.1)	25.2 (22.3 – 29.4)	24.5 (22.7 – 27.2)	25.0 (22.3 –28.5)
N/A	0 (0%)	1 (1.5%)	26 (6.5%)	2 (2.2%)	29 (5.1%)
BMI					
<18.8	0 (0%)	2 (3.0%)	8 (2.0%)	2 (2.2%)	12 (2.1%)
>18.8–24.2	6 (54.5%)	31 (46.3%)	144 (36.3%)	37 (41.1%)	218 (38.6%)
>24.2–30	5 (45.5%)	20 (29.9%)	135 (34.0%)	36 (40.0%)	196 (34.7%)
>30	0 (0%)	13 (19.4%)	83 (20.9%)	13 (14.4%)	109 (19.3%)
N/A	0 (0%)	1 (1.5%)	27 (6.8%)	2 (2.2%)	30 (5.3%)
Any disease					
No	4 (36.4%)	38 (56.7%)	221 (55.7%)	64 (71.1%)	327 (57.9%)
Yes	7 (63.6%)	29 (43.3%)	176 (44.3%)	26 (28.9%)	238 (42.1%)
Gestational diabetes					
No	9 (81.8%)	60 (89.6%)	346 (87.2%)	87 (96.7%)	502 (88.8%)
Yes	2 (18.2%)	6 (9.0%)	29 (7.3%)	1 (1.1%)	38 (6.7%)
N/A	0 (0%)	1 (1.5%)	22 (5.5%)	2 (2.2%)	25 (4.4%)
Heart disease					
No	11 (100%)	66 (98.5%)	367 (92.4%)	86 (95.6%)	530 (93.8%)
Yes	0 (0%)	0 (0%)	8 (2.0%)	2 (2.2%)	10 (1.8%)
N/A	0 (0%)	1 (1.5%)	22 (5.5%)	2 (2.2%)	25 (4.4%)
Epilepsy					
No	10 (90.9%)	65 (97.0%)	375 (94.5%)	88 (97.8%)	538 (95.2%)
Yes	1 (9.1%)	1 (1.5%)	0 (0%)	0 (0%)	2 (0.4%)
N/A	0 (0%)	1 (1.5%)	22 (5.5%)	2 (2.2%)	25 (4.4%)
Hypertension					
No	10 (90.9%)	66 (98.5%)	371 (93.5%)	87 (96.7%)	534 (94.5%)
Yes	1 (9.1%)	0 (0%)	3 (0.8%)	1 (1.1%)	5 (0.9%)
N/A	0 (0%)	1 (1.5%)	23 (5.8%)	2 (2.2%)	26 (4.6%)
Other diseases					
No	7 (63.6%)	42 (62.7%)	237 (59.7%)	67 (74.4%)	353 (62.5%)
Yes	4 (36.4%)	25 (37.3%)	160 (40.3%)	23 (25.6%)	212 (37.5%)
Medication					
No	3 (27.3%)	38 (56.7%)	219 (55.2%)	47 (52.2%)	307 (54.3%)
Yes	8 (72.7%)	28 (41.8%)	152 (38.3%)	41 (45.6%)	229 (40.5%)
N/A	0 (0%)	1 (1.5%)	26 (6.5%)	2 (2.2%)	29 (5.1%)
Vaccine doses					
0	11 (100%)	0 (0%)	397 (100%)	0 (0%)	408 (72.2%)
1	0 (0%)	4 (6.0%)	0 (0%)	14 (15.6%)	18 (3.2%)
2	0 (0%)	49 (73.1%)	0 (0%)	63 (70.0%)	112 (19.8%)
3	0 (0%)	14 (20.9%)	0 (0%)	13 (14.4%)	27 (4.8%)

Mothers at 12M follow-up	Infected		Not infected		Total
	Not vaccinated	Vaccinated	Not vaccinated	Vaccinated	(N=157)
	(N=9)	(N=115)	(N=2)	(N=31)	
Vaccines doses					
0	9 (100%)	0 (0%)	2 (100%)	0 (0%)	11 (7.0%)
1	0 (0%)	4 (3.5%)	0 (0%)	0 (0%)	4 (2.5%)
2	0 (0%)	90 (78.3%)	0 (0%)	23 (74.2%)	113 (72.0%)
3	0 (0%)	21 (18.3%)	0 (0%)	8 (25.8%)	29 (18.5%)
Duration of breastfeeding					
26 weeks or more	7 (77.8%)	81 (70.4%)	0 (0%)	25 (80.6%)	113 (72.0%)
Less than 26 weeks	1 (11.1%)	20 (17.4%)	2 (100%)	5 (16.1%)	28 (17.8%)
Never breastfeed	0 (0%)	3 (2.6%)	0 (0%)	1 (3.2%)	4 (2.5%)
N/A	1 (11.1%)	11 (9.6%)	0 (0%)	0 (0%)	12 (7.6%)
Continued breastfeeding at 12M follow-up					
Yes	6 (66.7%)	56 (48.7%)	2 (100%)	18 (58.1%)	82 (52.2%)
No	2 (22.2%)	48 (41.7%)	0 (0%)	13 (41.9%)	63 (40.1%)
N/A	1 (11.1%)	11 (9.6%)	0 (0%)	0 (0%)	12 (7.6%)

N/A refers to missing data.

Demographic data and characteristics of the children are depicted in **Table 2**. The study included 537 children with umbilical blood drawn after delivery. The median length of gestation within the cohort was 40.4 weeks (IQR: 39.6–41.3) and there was an even distribution of boys (50.7%) and girls (43.8%) among the children born. The median birth weight of the children was 3790 g (IQR: 3430 – 4110) and most of the children were delivered through vaginal birth (75.6%). Median neonatal APGAR scores after 1 and 5 minutes are provided in **Table 2**.

**Table 2:** Demographic data and characteristics of neonates.

Children at birth	Infected mother		Not infected mother		Total
	Not vaccinated mother	Vaccinated mother	Not vaccinated mother	Vaccinated mother	(N=537)
	(N=14)	(N=62)	(N=348)	(N=113)	
Gestation time (weeks)					
median (IQR)	40.0 (39.8 – 40.6)	40.1 (39.2 – 40.9)	40.6 (39.7 – 41.3)	40.3 (39.4 – 41.2)	40.4 (39.6 – 41.3)
N/A	0 (0%)	3 (4.8%)	21 (6.0%)	5 (4.4%)	29 (5.4%)
Sex					
Boy	6 (42.9%)	35 (56.5%)	173 (49.7%)	58 (51.3%)	272 (50.7%)
Girl	8 (57.1%)	24 (38.7%)	152 (43.7%)	51 (45.1%)	235 (43.8%)
N/A	0 (0%)	3 (4.8%)	23 (6.6%)	4 (3.5%)	30 (5.6%)
Birth weight (g)					
median (IQR)	3660 (3480 – 4260)	3800 (3290 – 4040)	3800 (3440 – 4120)	3770 (3450 – 4130)	3790 (3430 – 4110)
N/A	0 (0%)	3 (4.8%)	23 (6.6%)	5 (4.4%)	31 (5.8%)
Birth weight (g)					
<3445	3 (21.4%)	18 (29.0%)	85 (24.4%)	27 (23.9%)	133 (24.8%)
>3445–4090	7 (50.0%)	27 (43.5%)	152 (43.7%)	52 (46.0%)	238 (44.3%)
>4090	4 (28.6%)	14 (22.6%)	88 (25.3%)	29 (25.7%)	135 (25.1%)
N/A	0 (0%)	3 (4.8%)	23 (6.6%)	5 (4.4%)	31 (5.8%)
Delivery					
Caesarean section	1 (7.1%)	8 (12.9%)	70 (20.1%)	21 (18.6%)	100 (18.6%)
Vaginal	13 (92.9%)	51 (82.3%)	255 (73.3%)	87 (77.0%)	406 (75.6%)
N/A	0 (0%)	3 (4.8%)	23 (6.6%)	5 (4.4%)	31 (5.8%)
APGAR score 1min					
<8	1 (7.1%)	1 (1.6%)	24 (6.9%)	5 (4.4%)	31 (5.8%)
≥8	13 (92.9%)	58 (93.5%)	300 (86.2%)	102 (90.3%)	473 (88.1%)
N/A	0 (0%)	3 (4.8%)	24 (6.9%)	6 (5.3%)	33 (6.1%)
APGAR score 5min					
<8	0 (0%)	1 (1.6%)	13 (3.7%)	2 (1.8%)	16 (3.0%)
≥8	14 (100%)	58 (93.5%)	320 (92.0%)	106 (93.8%)	487 (90.7%)
N/A	0 (0%)	3 (4.8%)	25 (7.2%)	6 (5.3%)	34 (6.3%)

N/A refers to missing data.

### Detection of total anti-RBD and anti-N Ig in maternal and neonatal serum

**Figure 1:**
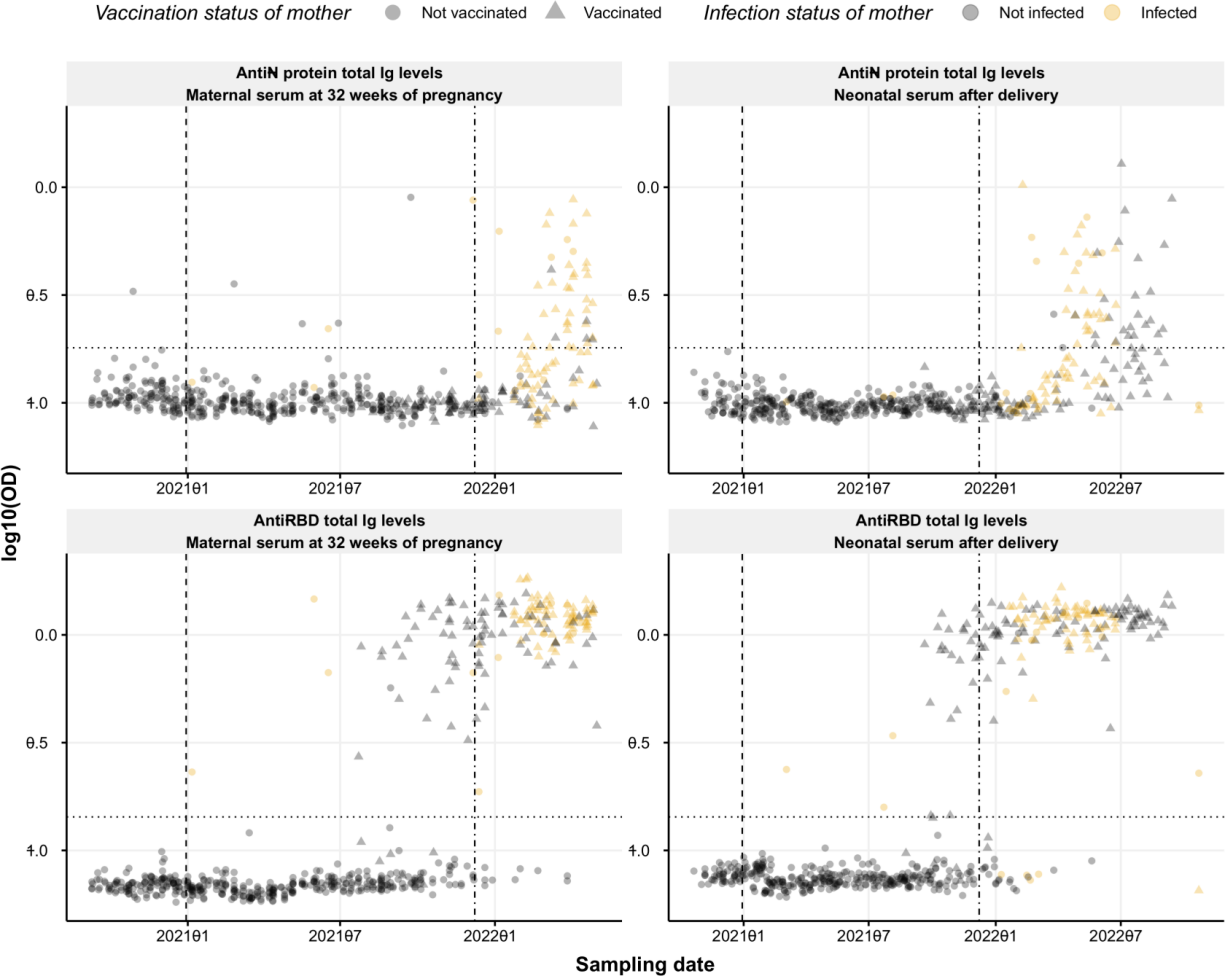
Seroprevalence of SARS-CoV-2 anti-RBD and anti-N protein Ig. Levels of RBD- and N protein-specific Ig (bottom and top panel, respectively) over time in maternal serum at 32 weeks of pregnancy (left) and neonatal cord serum right after delivery (right). Circles and triangles represent the observed levels of circulating Ig antibodies in non-vaccinated and vaccinated mothers, respectively. Grey and yellow colors represent not infected and infected mothers, respectively. The horizontal black dotted line represents the threshold for positivity in each assay. Vertical dashed and dash-dotted lines indicate vaccine rollout and omicron breakout in the Faroe Islands, respectively. Data are represented in log10(OD) and the horizontal line represents the threshold for assay positivity.

The levels of SARS-CoV-2-specific antibodies were evaluated using our semi-quantitative in-house developed sandwich ELISAs detecting total Ig against spike RBD or N protein. Seropositivity within the cohort relies on the time point of maternal or neonatal blood collection (**Figure 1**). Samples collected at the beginning of the study period remained seronegative for both RBD- and N-specific antibodies until the vaccine rollout and the emergence of the Delta and Omicron variant outbreak in the Faroe Islands. The first vaccination was given in the Faroe Islands on December 30, 2020. Still, no rise in the RBD-specific response was observed in maternal serum until summer 2021, when the recommendations changed regarding pregnant women and vaccination (14). In addition, there was a significant increase in the incidence of infection in the Faroe Islands during the Delta and Omicron outbreaks in late 2021 and the beginning of 2022 (15, 16). This fits with the rise in the N-specific response from December 2021 (**Figure 1**).

**Figure 2:**
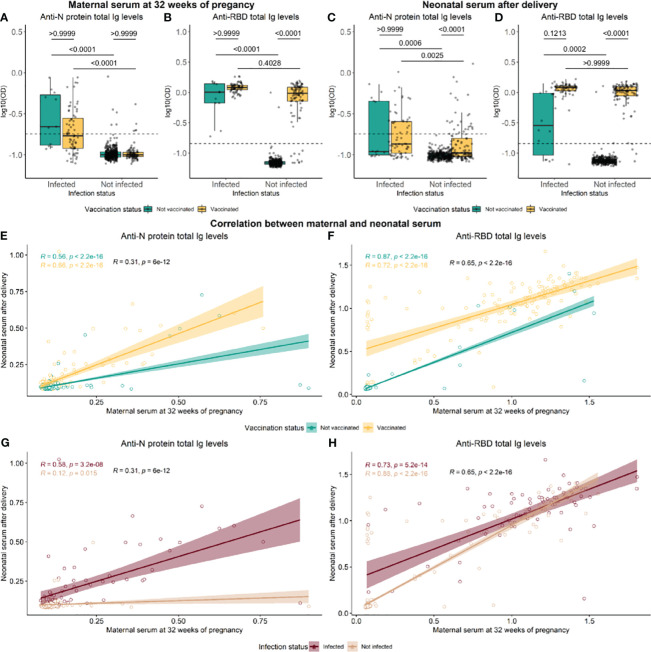
Distribution of antibodies against RBD and N-protein in maternal and neonatal serum. Levels of RBD- and N protein-specific in maternal serum at 32 weeks of pregnancy **(A, B)** and neonatal cord serum right after delivery **(C, D)**. Green and yellow colors represent not vaccinated and not vaccinated, respectively. Data reported as the median and interquartile range (box), whiskers represent 1.5 times the IQR. P-values were calculated using the Kruskal–Wallis test followed by multiple comparisons with Dunn’s correction. Spearman rank correlation of RBD and N protein Ig levels between maternal and neonatal serum, in groups of vaccinated **(E, F)** and infected individuals **(G, H)**. Data are represented in log10(OD) and the horizontal line represents the threshold for assay positivity. P-values < 0.05 were considered statistically significant.

In maternal serum, the levels of N-specific Ig were significantly different between individuals infected and not infected (p-value <0.0001). Still, no significance was found between vaccinated and not vaccinated individuals (p-value >0.9999) (**Figure 2A**). On the other hand, significant differences in RBD-specific Ig levels were found to be affected by both infection and vaccination status (p-value <0.0001) (**Figure 2B**). In neonates’ umbilical blood, both RBD and N protein-specific Ig levels were significantly affected by maternal infection and vaccination status (p-value <0.001) (**Figures 2C, D**). A significant correlation was found between RBD levels in maternal blood drawn at 32 weeks of pregnancy and neonatal umbilical cord blood collected at birth (rho=0.65, p-value <2.2e−16) (**Figures 2F, H**). The N-specific Ig levels correlated poorly, although significantly, between mothers and children (rho= 0.31, p-value <6e−12) (**Figures 2E, G**).

### Persistence of RBD- and N-specific levels after 12 months

**Figure 3:**
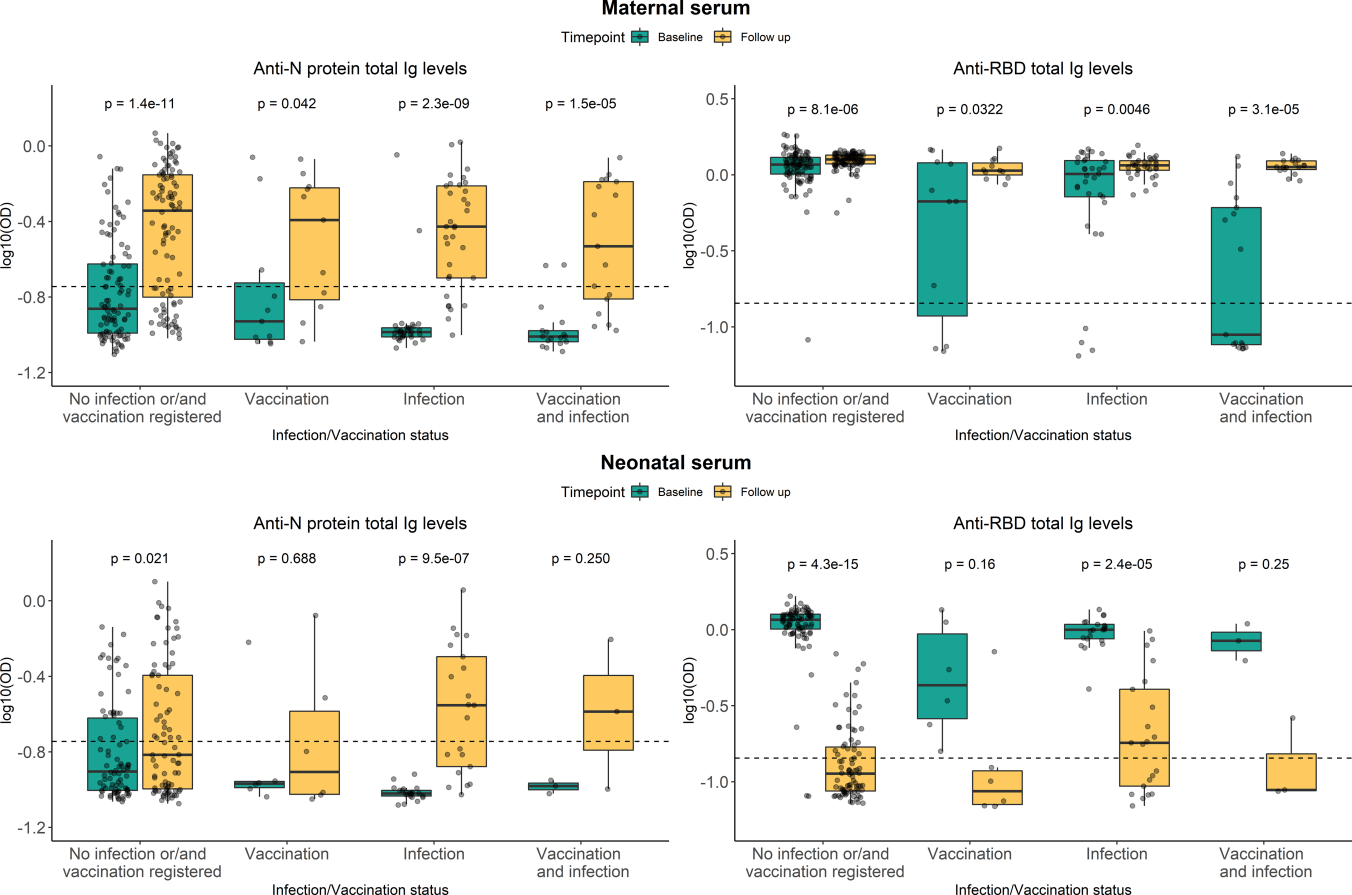
Persistence of RBD- and N-specific levels after 12 months. Levels of RBD- and N protein-specific Ig in maternal serum (top panel) and neonatal serum (bottom panel). Green and yellow colors represent baseline sampling and sampling at follow-up examination, respectively. Subdivided into groups based on maternal vaccination and/or infection during the period between baseline and follow-up sampling. Data reported as the median and interquartile range (box), whiskers represent 1.5 times the IQR. Data are represented in log10(OD) and the horizontal line represents the threshold for assay positivity. P-values < 0.05 were considered statistically significant by the Wilcoxon paired test.

To investigate the dynamics over time of the SARS-CoV-2 specific antibodies in neonatal and maternal serum all individuals with either a positive RBD- or N-specific response at baseline were selected for further analyses. These analyses included blood samples from 138 children and 157 mothers (105 were paired samples), all with samples collected at baseline and 12-month follow-up examinations. The resulting data is presented in **Figure 3**. In the figure, the women are subdivided into groups based on whether they were vaccinated and/or infected between baseline and follow-up sampling. Even in women without a registered vaccine- or infection-based immune boost, the median levels of antibodies against N protein significantly increased after 12 months (p-value = 1.4e−11) (**Figure 3** top-left). A similar tendency was seen for N-specific Ig levels in serum from children, although with lower levels of N-specific Ig in general (p-value = 0.021) (**Figure 3** bottom-left). The levels of RBD-specific Ig in maternal blood appeared to remain stable after 12 months, although significantly different (p-value = 8.1e−06) (**Figure 3** top-right), compared to children in which the RBD-specific Ig levels were significantly decreased at follow-up sampling (p-value = 4.3e−15) (**Figure 3** bottom-right).

**Figure 4:**
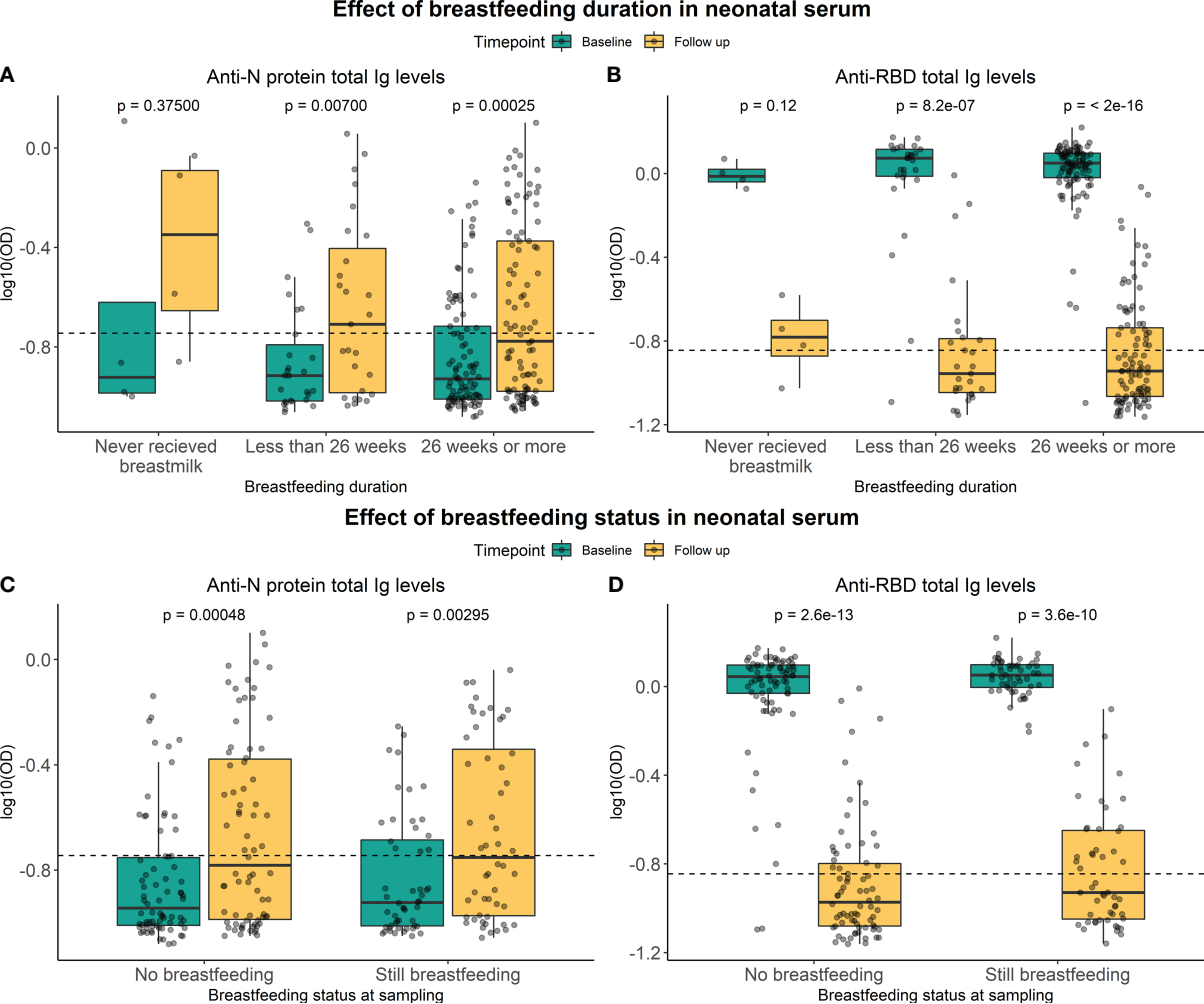
Effect of breastfeeding on RBD- and N-specific levels. Levels of RBD- and N protein-specific Ig in neonatal serum at baseline sampling (green) and follow-up sampling (yellow). They were subdivided into groups based on the duration of breastfeeding **(A, B)** and whether a child is still breastfed or not **(C, D)**. Data reported as the median and interquartile range (box), whiskers represent 1.5 times the IQR. Data are represented in log10(OD) and the horizontal line represents the threshold for assay positivity. P-values < 0.05 were considered statistically significant by the Wilcoxon paired test.

Whether total Ig levels in children’s serum after 12 months were indirectly affected by breastfeeding was addressed by subdividing children into groups based on how long the child had received breastmilk and whether they still received breastmilk at 12-month follow-up sampling (**Figure 4**). No significant difference was seen between the groups regarding the duration of breastfeeding for RBD or N protein (p-value = 0.5429 and p-value = 0.3027, respectively) (**Supplementary Table S1**). Furthermore, no significant difference was seen in Ig responses between children receiving breastmilk 12 months after delivery and those not receiving it for RBD or N protein (P-value = 0.0955 and p-value = 0.7879, respectively). The effect of breastfeeding was additionally tested using multiple linear regression models, confirming that no significant associations were found between the SARS-CoV-2-specific Ig levels at follow-up and breastfeeding (**Supplementary Table S2**). This indicated that breastfeeding had no impact on the persistence of SARS-CoV-2 specific antibody levels in serum from children one year after delivery.

### Assessment of immunoglobulin isotypes

All seropositive samples from the total Ig RBD sandwich assay were additionally analyzed in our direct setup to quantify the RBD-specific IgM, IgG, and IgA levels. The distribution of Ig isotypes in blood collected at baseline and follow-up are presented in **Supplementary Figures S3** and **S4**, respectively. Detectable levels of primarily IgG, IgM, and IgA were found in maternal blood at baseline (**Supplementary Figures S3A–C**). RBD-specific IgG levels in neonate serum collected at birth were significantly correlated with maternal IgG levels at baseline (rho=0.769, p-value <0.0001). Very few children had borderline measurable IgM or IgA-specific antibodies at delivery (**Supplementary Figures S3D–F**). All mothers had acquired a relatively high and stable RBD-specific IgG response at 12 months follow-up and generally low detectable levels of IgM and IgA (**Supplementary Figures S4A–C**). At the time of sample collection, 93% of the mothers were vaccinated, and 79% had had a COVID-19 infection before follow-up blood collection. However, children’s levels of IgG were close to the detection limit, in addition to a few children with a borderline positive signal for IgM in blood collected after 12 months (**Supplementary Figures S4D–F**).

### Maternal influence on neonatal antibody levels and pregnancy outcome

Multiple linear regression models were used to address the effect of maternal parameters on pregnancy outcomes. First, we performed an analysis investigating whether the mother’s demographic variables (age, BMI, comorbidities, use of medication, vaccination- and infection status of mother) were associated with anti-RBD and anti-N protein responses in maternal and neonate serum at baseline (**Table 3**). The models used for studying antibody levels in maternal serum were adjusted for age and BMI, while those used for studying antibody levels in neonatal serum were unadjusted. Maternal RBD Ig levels were positively associated with vaccination- and infection status (p-value <2e-16 for both), while maternal N protein Ig levels were associated with infection status only (p-value <2e-16). In neonatal serum sampled at delivery, the RBD- and N protein-specific Ig levels were associated with both the vaccination and infection status of the mothers (**Table 3**). Increasing maternal age was found to be negatively associated with N-specific antibody levels in neonate serum (p-value = 0.0397). In contrast, maternal comorbidities were found to be negatively associated with neonatal anti-RBD levels (p-value <0.0001). Finally, when studying whether maternal parameters were associated with RBD- and N-specific Ig levels in children’s blood collected at follow-up examination after 12 months, no associations with maternal parameters were found (**Supplementary Table S1**).

**Table 3:** Linear regression models investigating associations between maternal parameters and the SARS-CoV-2 Ig levels in maternal and neonatal serum.

	Maternal serum at 32 weeks of pregnancy^a^				Neonatal serum at birth^b^			
	RBD Ig levels		N protein Ig levels		RBD Ig levels		N protein Ig levels	
	EST	p-value	EST	p-value	EST	p-value	EST	p-value
**Age**	-0.0035	0.4331	0.0002	0.8150	-0.0036	0.4186	-0.0018	0.0397*
**BMI**	-0.0020	0.6182	-0.0014	**0.0959**	-0.0053	0.1770	-0.0012	0.1260
**Comorbidities**	-0.0604	0.1612	0.0079	0.3670	-0.1690	0.0001***	-0.0187	**0.0618**
Medication^c^	0.0254	0.5617	0.0140	0.1168	0.0639	0.1410	0.0011	0.9020
**Vaccination status**	0.7516	<2e-16***	-0.0131	0.1683	0.9470	< 2e-16***	0.0779	1.26e-14***
**Infection status**	0.4475	<2e-16***	0.1565	2e-16***	0.7383	< 2e-16***	0.1084	1.26e-15***

a adjusted for age and BMI using linear regression.

b unadjusted using linear regression.

c intake during pregnancy.

P-values < 0.05 were considered statistically significant. Values significant or borderline significant are highlighted in bold. Significance levels are *p < 0.05, **p < 0.01, and ***p < 0.001.

The regression models were then applied to study the association between vaccination and/or infection status of the mother, and thereby also SARS-CoV-2 specific antibody responses, with the pregnancy outcome (**Table 4**). All models were adjusted for at least maternal age and BMI, as indicated in the table. A negative association was found between gestation weeks and maternal age (p-value = 0.0118). Interestingly, a positive association was found with gestation weeks for mother vaccination status and RBD Ig levels (p-value = 0.0397 and p-value = 0.0177, respectively). Birth weight and delivery were positively associated with maternal BMI (p-value = 0.0023 and p-value = 0.0003, respectively). Neonatal 1-minute and 5-minute APGAR scores were negatively associated with the age of the mothers (p-value = 0.0080 and p = 0.0016, respectively). Finally, a positive association was found between neonatal 1-minute APGAR score and maternal vaccination status (p-value = 0.0048) and RBD Ig levels (p-value = 0.0236).

**Table 4:** Linear and logistic regression models investigating associations between maternal parameters and the pregnancy outcome.

	Weeks of gestation^a^		Birth weight^b^		Delivery^c^		Apgar score 1 min^d^		Apgar score 5 min^d^	
	EST	p-value	EST	p-value	EST	p-value	EST	p-value	EST	p-value
**Age**	-0.0304	0.0118*	-0.3308	0.9394	-0.0411	0.1031	-0.0307	0.0080**	-0.0152	0.0016**
**BMI**	-0.0168	0.1189	11.730	0.0023**	-0.0747	0.0003***	0.0089	0.3845	-0.0007	0.8603
**Comorbidities**	-0.2012	**0.0841**	9.2222	0.8265	0.1848	0.4536	0.1586	0.155	0.0651	0.1587
**Medication^e^**	-0.1392	0.2561	5.3466	0.9016	0.0339	0.8927	-0.0574	0.6170	-0.0108	0.8212
**Vaccination status**	-0.2704	0.0397*	8.6571	0.8573	0.4533	0.1381	0.3599	0.0048**	0.0574	0.2788
**Infection status**	-0.2772	0.1262	-25.766	0.6934	0.5748	0.2097	0.2036	0.2407	0.0429	0.5507
**RBD Ig levels**	-0.2948	0.0177*	-19.032	0.6722	0.3033	0.2810	0.2696	0.0236*	0.0642	0.19407
**N protein Ig levels**	0.5237	0.4156	-50.603	0.8267	-0.3454	0.7916	0.8245	0.1797	0.1978	0.4369

a adjusted for age, BMI, and comorbidities using linear regression.

b adjusted for age, BMI, and weeks of gestation using linear regression.

c adjusted for age and BMI using logistic regression.

d adjusted for age and BMI using linear regression.

e intake during pregnancy.

P-values < 0.05 were considered statistically significant. Values significant or borderline significant are highlighted in bold. Significance levels are *p < 0.05, **p < 0.01, and ***p < 0.001.

### The variant specificity of the RBD-specific antibodies

**Figure 5:**
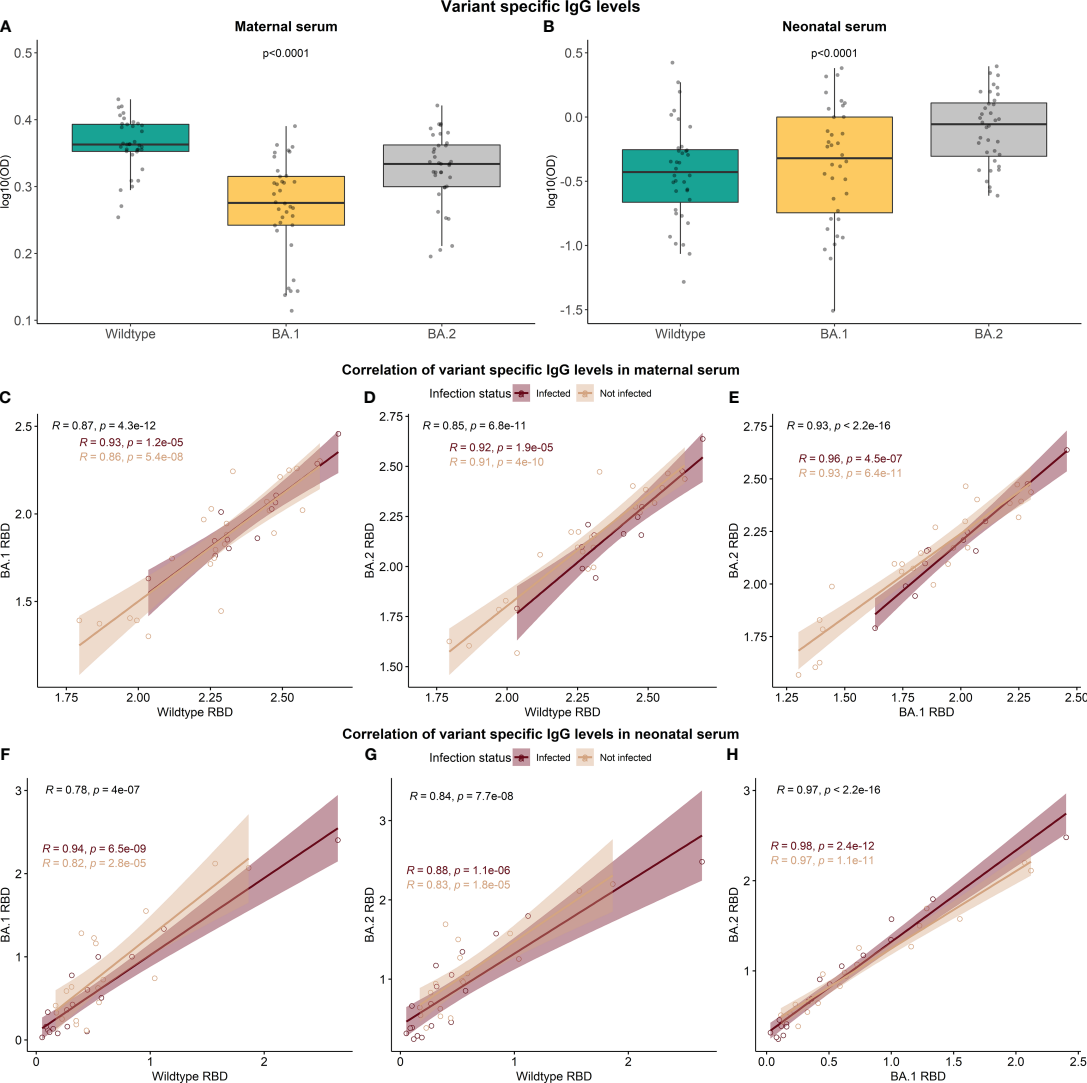
The variant specificity of the RBD-specific antibodies. A total of 36 sample pairs from mother and child collected at the 12-month follow-up (n=72) were analyzed in a direct setup detecting IgG antibodies against ancestral RBD (Wildtype) and two Omicron variants, B.1.1.529 RBD and BA.2 RBD **(A, B)**. Data reported as the median and interquartile range (box), whiskers represent 1.5 times the IQR. Data are represented in log10(OD) and p-values < 0.05 were considered statistically significant by the Friedman test. Spearman rank correlation of RBD IgG levels between the variants was performed **(C–H)**.

At follow-up examination, some children had detectable N-specific antibodies but were seronegative for antibodies against RBD. Our RBD assays utilize ancestral RBD, while the main cause of SARS-CoV-2 infection in the Faroe Islands was the Omicron variant B1.1.529 and BA.2 (17). It, therefore, became of interest to investigate the specificity of the RBD-specific antibodies towards different SARS-CoV-2 RBD variants to elucidate a potential explanation for the high proportion of non-detectable RBD-specific antibodies in children’s serum after 12 months. Samples from a total of 36 paired mothers and children from the 12-month follow-up were included in the analysis; 18 mother-child pairs in which the child had a negative response towards RBD but a positive response towards N protein, and 18 mother-child pairs in which the child has a positive response to both RBD and N protein. All 72 samples were analyzed in a direct ELISA setup detecting IgG antibodies on a coat of ancestral RBD (Wildtype) or the Omicron variants B.1.1.529 RBD and BA.2 RBD. The resulting data indicate that there is a significant difference between RBD-specific IgG depending on the variant-specific antigen employed for both mothers (p-value <0.0001) and children (p-value <0.0001) (**Figures 5A, B**). However, the overall distribution of variant-specific IgG levels varies between mothers and children. In maternal serum, the median levels of antibodies against Wildtype RBD and BA.2 RBD were higher compared to the B.1.1.529 variant. In contrast, the median levels of antibodies against BA.2 RBD in neonatal serum significantly increased compared to the remaining two variants. However, the IgG levels in children were generally lower than in mothers for all three variants studied. The levels of RBD-specific IgG were highly correlated between the variants in maternal and neonatal serum (**Figures 5C–H**).

## Discussion

Neonates exhibit a limited ability to mount their immune responses, due to an immature/developing immune system (2). The maternal antibodies, mainly IgG, that are transferred across the placenta during pregnancy, provide vital protection against infections during the early months of life (18). Therefore, infant antibody responses to viral infections differ from adults and are highly dependent on the passive antibody transfer for bolstering the neonate’s immune defenses until their immune system matures. Studies describing the characteristics of the SARS-CoV-2 antibody response in neonates and direct comparisons between neonate and maternal antibody levels can be found in the literature. However, this data is often limited by low sample counts and a lack of long-term follow-up sampling. We therefore sought to investigate COVID-19 immunity in a large population-based prospective cohort, including neonates and paired mothers from the Faroe Islands. The data show that maternal IgG against SARS-CoV-2 is vertically transferred to the fetus during pregnancy. This is in agreement with the literature, in which several studies have shown that SARS-CoV-2 spike-specific IgG is transferred across the placenta and provides passive immunity to neonates (19, 20). Nevertheless, our observations are among a few studies that have confirmed vertical transfer of N-specific antibodies to the neonate circulation and demonstrated detectable levels of N protein Ig in umbilical cord blood after delivery (21, 22).

It has been suggested that the inflammatory response induced by SARS‐CoV‐2 may damage the placenta of pregnant women, raising the probability of viral vertical transmission. The inflammatory environment in the mother may be extended to the fetus, leading to compromise in its normal development (23). However, it is generally accepted that the risk of a mother passing on a COVID-19 infection to her unborn baby is low. This was recently confirmed in a review analyzing 244 placentas from women with COVID-19 during pregnancy, providing evidence of low risk of vertical transmission to the neonate (24). Placental and fetal infections have only been reported in a few studies, and data on vertical transmission are often inconclusive due to false‐positive testing or the fact that postnatal infection cannot be excluded (25). Almost no neonates in our study had measurable IgM or IgA-specific antibodies at birth, which aligns with existing knowledge regarding the vertical transfer of only IgG antibodies across the placenta (26). Three infants were positive for IgM in umbilical blood, suggesting either the rare intrauterine transmission of infection to the fetus or false‐positive testing. Although uncommon, IgM in the cord blood could also be secondary to placental microvascular injury and “leakage” of maternal IgM into fetal circulation (27).

Utilizing two in-house-produced SARS-CoV-2 assays for the detection of RBD and N protein-specific antibodies has enabled us to analyze and discriminate between vaccine- and infection-induced immunity since all current vaccinations in Europe are directed against the spike protein. As described in previous publications, both assays perform with high sensitivity and specificity (11, 12). The data presented in this study indicates maternal vaccination and infection status highly affect SARS-CoV-2 specific Ig levels in umbilical cord blood. A recent study investigated the antibody functions in paired maternal and cord blood to understand the implications of vaccination and infection during pregnancy concerning neonatal immunity. They found that maternal vaccination, compared to infection, had a higher impact on fetal antibody functional potency (28). Another study found that maternal and neonate cord blood IgG antibody levels were higher after SARS-CoV-2 vaccination than after SARS-CoV-2 infection (29). It is generally accepted that SARS-CoV-2 vaccination before or during pregnancy effectively and safely raises SARS-CoV-2-specific antibody titers in maternal and neonatal blood (30, 31). One study found that a 2-dose vaccination series administered during gestation led to an appreciable RBD-specific IgG response in maternal circulation, umbilical cord blood, neonate blood, and breast milk (32). In our study, the RBD-specific antibody response at baseline correlates better between the mother and neonate than the N protein-specific response. It is, however, important to remember that maternal blood was collected at 32 weeks of pregnancy, while neonatal blood was collected from the umbilical cord at delivery – leaving a gap of approximately 8 weeks, preventing a direct comparison between neonatal and maternal Ig levels at delivery. A portion of the mothers were likely infected during this in-between period since more than 13,000 cases were registered from September 1, 2021; to January 24, 2022; during the Delta and subsequently Omicron outbreak in the Faroe Islands (33).

The persistence of the maternally derived anti-SARS-CoV-2 IgG is poorly understood, but a recent study found that infants continued to have functional maternal vaccine-derived anti-SARS-CoV-2 antibodies up to 12 weeks of age (34). Other studies have found the persistence of anti-SARS-CoV-2 IgG antibodies in infants, both after natural infection and from maternal vaccination during pregnancy, for up to 6 months (8, 20, 35). Our study showed a significant increase in median N-specific antibody levels in maternal and children’s serum after 12 months, even in women without a registered vaccine- or infection-based immune boost. The increase of maternal N-specific Ig is presumed due to asymptomatic infections and/or lack of RT-PCR testing, which was ceased on 1 March 2023 in the Faroe Islands. A reasonable explanation for an increase in children’s N-specific antibody levels could likewise be that some children become infected with SARS-CoV-2 during the first year of their life. Children are expected to be able to produce their antibodies from approximately 6 months of age (36), and production of SARS-CoV-2-specific Ig has been observed from early infancy (37). However, we also observed a substantial decrease in RBD-specific antibodies in children’s blood after 12 months. We would have expected RBD-specific levels to be equally increased as for N protein antibodies, at least concerning what we know about antibody responses to COVID-19 infection in adults (38). It is important to highlight that we have not been able to discriminate between antibodies transferred from the mother to the infant and the infant’s antibody response to SARS-CoV-2 upon infection, or both. Several children with a blood sample collected at 12-month examination have RBD-specific antibodies below cut-off while being seropositive for N-specific Ig. We speculated if the lack of RBD-specific antibodies was due to the antibody assays being based on the original Wildtype RBD strain and, therefore, might not accurately capture infections with the Omicron variant in un-vaccinated children. The performed analysis with B.1.1529 and BA.2 RBD antigens showed highly correlated IgG levels between the variants, suggesting that using the Wildtype RBD strain as antigen still reflects genuine RBD-specific Ig levels, even after the introduction of Omicron. In support of this, a recent study has examined the humoral responses to SARS-CoV-2 infection in 23 infants/young children and likewise found striking differences in the durability of binding antibodies to SARS-CoV-2 spike and N antigens. They observed the same tendency: spike-specific responses rapidly declined in children while a comparable decline of N-specific IgG antibodies was observed between children and adults (37). This suggests that anti-spike and -N humoral responses may be differentially regulated, implying that the increased durability in children is not necessarily universal for every antigen. It has also been shown that the N protein is highly immunogenic, contributing to the difference in RBD and N-specific immune responses (39). Further investigations are needed to understand the potential differences in regulatory patterns of RBD- and N protein-specific antibodies in neonates. Finally, human milk offers passive immunity to the breastfed infant by transferring disease-specific antibodies, mostly IgA (40). The same has been observed for SARS-CoV-2-specific antibodies (41) and a recent study has likewise proposed that breastmilk are conferring immunity to infants against COVID-19 (42). However, the extent of breastmilk-derived protection against COVID-19 and how this relates to the antibodies residing in fetal blood remains to be elucidated. We were interested in assessing the indirect impact of breastfeeding on the newborn’s capacity to generate an immune response. We found no significant association of breastfeeding with SARS-CoV-2-specific Ig levels in the present study.

Linear regression models were used to address the effect of maternal vaccine- and/or infection status on the pregnancy outcome. The variables applied to measure pregnancy outcome included gestational age, birth weight, delivery type, and 1- and 5-minute APGAR score. The analysis shows that gestational age is significantly negatively associated with maternal age, suggesting that women with high age have shorter pregnancies than younger mothers. This is in agreement with the literature (43). we also found that pre-pregnancy BMI was associated with neonatal birth weight. It is well known from the literature that fetuses of obese women have significantly higher weights than fetuses of nonobese women (44). Interestingly, gestational age was associated with the mother’s vaccination status and the RBD-specific Ig levels at 32 weeks of pregnancy, indicating that vaccinated women with high levels of anti-RBD have shorter pregnancies. However, no preterm neonates were included in this study, so “shorter” pregnancies here will refer to pregnancies still within the term. The proposed biological plausibility linking a mother’s vaccination status to pregnancy duration could be related to the potential impact of the immune response elicited by the vaccine. One potential mechanism is that the immune response triggered by vaccination may lead to a systemic inflammatory response, which could potentially influence factors involved in pregnancy, such as the timing of labor initiation. Inflammatory responses have been linked to the onset of labor, as certain immune molecules and signaling pathways play a role in the initiation of labor contractions (45, 46). It’s important to note that any potential effect of vaccination on pregnancy duration, if present, is likely to be minimal and not clinically meaningful. The degree of impact would likely be negligible relative to the complex network of factors that influence the timing and progression of labor and delivery. Vaccine status and increased maternal RBD-specific IgG levels were also associated with increased 1-minute APGAR scores after delivery. Whether the APGAR score can be associated with long-term developmental outcomes has been widely debated (47). However, several recent studies have found significant associations between lower APGAR scores and later-life complications (48, 49). Interestingly, another study likewise found that the COVID-19-Induced Immunity Response (CIIR) was significantly and inversely correlated with APGAR scores (50). We also found an association between 1- and 5-minute APGAR scores and maternal age, suggesting that women of higher age give birth to babies with significantly lower APGAR scores. It has been shown that older women (above 35 years) have higher odds of low APGAR scores than women aged 20–35 (51). Concerning maternal age, it is important to remember that we have a cohort of pregnant women with a narrow age span included in the study (median age: 31, IQR: 28–34). This is most likely the explanation as to why age was not associated with maternal SARS-CoV-2 Ig levels, which is widely accepted in the literature (12, 52).

Overall, the regression models provide indications of potential associations between maternal vaccination, RBD-specific antibody levels, and certain pregnancy outcomes. At least in adults, Spike and RBD levels are known to be highly associated with and driven by vaccination (53). This type of analysis is complicated by the many parameters that can affect neonatal immune responses, including maternal infection and/or vaccination and the timing. Many studies have investigated dose dependency and timing of immunization during pregnancy, which could be considered important regarding the transplacental transfer of protective antibodies to neonates (34). However, the conclusions are somehow contradicting. Most studies found a positive correlation between levels of spike-specific antibodies and gestational age of vaccination and that the correlation was specifically stronger after the second dose of vaccination (30). Contrary to this, other studies found no or a negative correlation between anti-spike IgG and vaccine timing during pregnancy (54–56). In addition, several other factors are known to significantly affect SARS-CoV-2 immune responses, such as COVID-19 disease severity (12, 57). Moreover, it is important to consider that immunity to SARS-CoV-2 extends beyond antibody responses (58). Further exploration into vaccine- and RBD association with pregnancy outcomes is needed to further elucidate what causes they could be attributed to.

In summary, the data presented in this study show that neonates have transplacental transferred levels of RBD- and N protein-specific SARS-CoV-2 antibodies, which correlate with the mother’s antibody levels and are influenced by the vaccine and infection status of the mother. However, anti-RBD and anti-N protein humoral responses seem to be differentially regulated in children compared to the mothers. Finally, we find an association between maternal vaccination and levels of SARS-CoV-2 specific antibodies against spike RBD with certain pregnancy outcomes, including gestational age and APGAR scores.

## Limitations of the study

This study presents some limitations, including the relatively small group sizes for both the vaccination and infection groups at baseline. The limited number of women who were infected and/or vaccinated introduces uncertainties when including these variables in the models due to a lack of statistical power. Moreover, since only baseline-positive individuals were included in the follow-up analysis, this cohort is also naturally limited in the number of samples. In addition, the study faces limitations in the discrimination between antibodies transferred from the mother to the infant and the infant’s antibody response to SARS-CoV-2 upon infection, or both. The lack of further phenotypic information about the cohort raises uncertainty about natural infection occurrences, exacerbated by a lengthy interval between birth and the 12-month follow-up. Finally, the clinical data can include possible bias as a result of the self-reported responses to the questionnaires.

## Data availability statement

The original contributions presented in the study are included in the article/**Supplementary Material**. Further inquiries can be directed to the corresponding author.

## Ethics statement

The studies involving humans were approved by The Faroese Research Ethical Committee. The studies were conducted in accordance with the local legislation and institutional requirements. Written informed consent for participation in this study was provided by the participants’ legal guardians/next of kin.

## Author contributions

IJ: Writing – original draft, Writing – review & editing, Conceptualization, Data curation, Formal analysis, Investigation, Methodology, Project administration, Software, Validation, Visualization. CH: Writing – original draft, Writing – review & editing, Conceptualization, Data curation, Formal analysis, Investigation, Methodology, Project administration, Software, Validation, Visualization. LP-A: Writing – original draft, Writing – review & editing, Conceptualization, Data curation, Formal analysis, Investigation, Methodology, Project administration, Software, Validation, Visualization. PW: Writing – original draft, Writing – review & editing, Conceptualization, Funding acquisition, Resources, Supervision. MP: Writing – original draft, Writing – review & editing, Conceptualization, Funding acquisition, Resources, Supervision. PG: Writing – original draft, Writing – review & editing, Conceptualization, Funding acquisition, Resources, Supervision.

## References

[1] World Health Organization. 2019 Coronavirus disease (COVID-19) pandemic. Geneva, Switzerland: World Health Organization. Available online at: https://www.who.int/emergencies/diseases/novel-coronavirus-2019 (Accessed October 18, 2023).

[2] Heinonen S Rodriguez-Fernandez R Diaz A Oliva Rodriguez-Pastor S Ramilo O Mejias A. Infant immune response to respiratory viral infections. Immunol Allergy Clin North Am. (2019) 39:361–76. doi: 10.1016/j.iac.2019.03.005 PMC6625527 31284926

[3] Devera JL Gonzalez Y Sabharwal V. A narrative review of COVID-19 vaccination in pregnancy and breastfeeding. J Perinatol Off J Calif Perinat Assoc. (2023) 44:12–9. doi: 10.1038/s41372-023-01734-0 37495712

[4] Liu S Zhong J Zhang D. Transplacental transfer of maternal antibody against SARS-CoV-2 and its influencing factors: A review. Vaccines. (2022) 10:1083. doi: 10.3390/vaccines10071083 PMC9318929 35891247

[5] Atyeo CG Shook LL Brigida S De Guzman RM Demidkin S Muir C. Maternal immune response and placental antibody transfer after COVID-19 vaccination across trimester and platforms. Nat Commun. (2022) 13:3571. doi: 10.1038/s41467-022-31169-8 35764643 PMC9239994

[6] Gray KJ Bordt EA Atyeo C Deriso E Akinwunmi B Young N. Coronavirus disease 2019 vaccine response in pregnant and lactating women: a cohort study. Am J Obstet Gynecol. (2021) 225:303.e1–303.e17. doi: 10.1016/j.ajog.2021.03.023 PMC7997025 33775692

[7] Prabhu M Murphy EA Sukhu AC Yee J Singh S Eng D. Antibody response to coronavirus disease 2019 (COVID-19) messenger RNA vaccination in pregnant women and transplacental passage into cord blood. Obstet Gynecol. (2021) 138:278–80. doi: 10.1097/AOG.0000000000004438 PMC8288193 33910219

[8] Shook LL Atyeo CG Yonker LM Fasano A Gray KJ Alter G. Durability of anti-spike antibodies in infants after maternal COVID-19 vaccination or natural infection. JAMA. (2022) 327:1087–9. doi: 10.1001/jama.2022.1206 PMC8822441 35129576

[9] Wimmers F Burrell AR Feng Y Zheng H Arunachalam PS Hu M. Multi-omics analysis of mucosal and systemic immunity to SARS-CoV-2 after birth. Cell [Internet]. (2023) 186:4632–51. doi: 10.1016/j.cell.2023.08.044 PMC10724861 37776858

[10] Burns MD Muir C Atyeo C Davis JP Demidkin S Akinwunmi B. Relationship between anti-spike antibodies and risk of SARS-CoV-2 infection in infants born to COVID-19 vaccinated mothers. Vaccines. (2022) 10:1696. doi: 10.3390/vaccines10101696 PMC9610427 36298561

[11] Jarlhelt I Pérez-Alós L Bayarri-Olmos R Hansen CB Petersen MS Weihe P. Distinguishing SARS-CoV-2 infection and vaccine responses up to 18 months post-infection using nucleocapsid protein and receptor-binding domain antibodies. Microbiol Spectr [Internet]. (2023) 0:e01796–23. doi: 10.1128/spectrum.01796-23 PMC10580960 37738355

[12] Hansen CB Jarlhelt I Pérez-Alós L Hummelshøj Landsy L Loftager M Rosbjerg A. SARS-CoV-2 antibody responses are correlated to disease severity in COVID-19 convalescent individuals. J Immunol. (2021) 206:109–17. doi: 10.4049/jimmunol.2000898 33208457

[13] Pérez-Alós L Armenteros JJA Madsen JR Hansen CB Jarlhelt I Hamm SR. Modeling of waning immunity after SARS-CoV-2 vaccination and influencing factors. Nat Commun. (2022) 13:1–11. doi: 10.1038/s41467-022-29225-4 35347129 PMC8960902

[14] Covid-19 Maternal Immunization Tracker (COMIT). Available online at: https://www.comitglobal.org/organization/recEz0DxRkRwqrTTQ (Accessed October 23, 2023).

[15] World Health Organization. WHO Coronavirus (COVID-19) Dashboard. Geneva, Switzerland: World Health Organization. Available online at: https://covid19.who.int/ (Accessed January 13, 2024).

[16] Petersen MS Kongsstovu S Í Eliasen EH Larsen S Hansen JL Vest N. Clinical characteristics of the Omicron variant - results from a Nationwide Symptoms Survey in the Faroe Islands. Int J Infect Dis IJID Off Publ Int Soc Infect Dis. (2022), 122:636–43. doi: 10.1016/j.ijid.2022.07.005 PMC9303132 35811082

[17] Petersen MS Pérez-Alós L Í Kongsstovu SK Eliasen EH Hansen CB Larsen S. Diverging humoral and cellular immune responses due to Omicron—a national study from the Faroe Islands. Microbiol Spectr. (2023) 11:e0086523. doi: 10.1128/spectrum.00865-23 PMC10714973 37909772

[18] Keller MA Stiehm ER. Passive immunity in prevention and treatment of infectious diseases. Clin Microbiol Rev. (2000) 13:602–14. doi: 10.1128/CMR.13.4.602 PMC88952 11023960

[19] Karimi H Mansouri V Rezaei N. Vertical transmission and maternal passive immunity post-SARS-CoV-2. Future Virol. (2023) 10.2217/fvl-2023-0089. doi: 10.2217/fvl-2023-0089 PMC10564388 37822684

[20] Song D Prahl M Gaw SL Narasimhan SR Rai DS Huang A. Passive and active immunity in infants born to mothers with SARS-CoV-2 infection during pregnancy: prospective cohort study. BMJ Open. (2021) 11:e053036. doi: 10.1136/bmjopen-2021-053036 PMC8264915 34234001

[21] Wachman EM Snyder-Cappione J Devera J Boateng J Dhole Y Clarke K. Maternal, infant, and breast milk antibody response following COVID-19 infection in early versus late gestation. Pediatr Infect Dis J. (2023) 42:e70–6. doi: 10.1097/INF.0000000000003802 PMC9935237 36729773

[22] Kashani-Ligumsky L Lopian M Cohen R Senderovich H Czeiger S Halperin A. Titers of SARS CoV-2 antibodies in cord blood of neonates whose mothers contracted SARS CoV-2 (COVID-19) during pregnancy and in those whose mothers were vaccinated with mRNA to SARS CoV-2 during pregnancy. J Perinatol. (2021) 41:2621–4. doi: 10.1038/s41372-021-01216-1 PMC8475451 34564695

[23] Fenizia C Biasin M Cetin I Vergani P Mileto D Spinillo A. Analysis of SARS-CoV-2 vertical transmission during pregnancy. Nat Commun [Internet]. (2020) 11:5128. doi: 10.1038/s41467-020-18933-4 PMC7552412 33046695

[24] Colley CS Hutchinson JC Whitten SM Siassakos D Sebire NJ Hillman SL. Routine placental histopathology findings from women testing positive for SARS-CoV-2 during pregnancy: Retrospective cohort comparative study. BJOG Int J Obstet Gynaecol. (2023) 130:959–67. doi: 10.1111/1471-0528.17476 37077035

[25] Edlow AG Li JZ Collier ARY Atyeo C James KE Boatin AA. Assessment of maternal and neonatal SARS-CoV-2 viral load, transplacental antibody transfer, and placental pathology in pregnancies during the COVID-19 pandemic. JAMA Netw Open. (2020) 3:e2030455. doi: 10.1001/jamanetworkopen.2020.30455 33351086 PMC7756241

[26] Albrecht M Arck PC. Vertically transferred immunity in neonates: Mothers, mechanisms and mediators. Front Immunol. (2020) 11. doi: 10.3389/fimmu.2020.00555 PMC7136470 32296443

[27] Flores-Pliego A Miranda J Vega-Torreblanca S Valdespino-Vázquez Y Helguera-Repetto C Espejel-Nuñez A. Molecular insights into the thrombotic and microvascular injury in placental endothelium of women with mild or severe COVID-19. Cells. (2021) 10:364. doi: 10.3390/cells10020364 PMC7916402 33578631

[28] Adhikari EH Lu P Kang YJ McDonald AR Pruszynski JE Bates TA. Diverging maternal and cord antibody functions from SARS-CoV-2 infection and vaccination in pregnancy. J Infect Dis. (2023) 229:462–72. doi: 10.1093/infdis/jiad421 PMC10873180 37815524

[29] Puopolo KM Flannery DD Gouma S Dhudasia MB Mukhopadhyay S Pfeifer MR. Comparison of Maternal and Neonatal Antibody Levels after COVID-19 Vaccination vs SARS-CoV-2 Infection. JAMA Netw Open. (2022) 5:e2240993. doi: 10.1001/jamanetworkopen.2022.40993 36350652 PMC9647482

[30] Marchand G Masoud AT Grover S King A Brazil G Ulibarri H. Maternal and neonatal outcomes of COVID-19 vaccination during pregnancy, a systematic review and meta-analysis. NPJ Vaccines. (2023) 8:1–9. doi: 10.1038/s41541-023-00698-8 37454153 PMC10349851

[31] Popescu DE Cîtu C Jura AMC Lungu N Navolan D Craina M. The Benefits of Vaccination against SARS-CoV-2 during Pregnancy in Favor of the Mother/Newborn Dyad. Vaccines. (2022) 10:1–11. doi: 10.3390/vaccines10060848 PMC9228905 35746456

[32] Marshall NE Blanton MB Doratt BM Malherbe DC Rincon M True H. SARS-CoV-2 vaccine booster elicits robust prolonged maternal antibody responses and passive transfer to the offspring *via* the placenta and breastmilk. Am J Obstet Gynecol MFM. (2023) 5:1–4. doi: 10.1016/j.ajogmf.2022.100830 PMC9710099 36462615

[33] Helmsdal G Hansen OK Møller LF Christiansen DH Petersen MS Kristiansen MF. Omicron outbreak at a private gathering in the Faroe Islands, infecting 21 of 33 triple-vaccinated healthcare workers. Clin Infect Dis. (2022) 75:893–6. doi: 10.1093/cid/ciac089 PMC9383377 35134167

[34] Prahl M Golan Y Cassidy AG Matsui Y Li L Alvarenga B. Evaluation of transplacental transfer of mRNA vaccine products and functional antibodies during pregnancy and infancy. Nat Commun. (2022) 13:1–12. doi: 10.1038/s41467-022-32188-1 35908075 PMC9338928

[35] Cambou MC Liu CM Mok T Fajardo-Martinez V Paiola SG Ibarrondo FJ. Longitudinal evaluation of antibody persistence in mother-infant dyads after severe acute respiratory syndrome coronavirus 2 infection in pregnancy. J Infect Dis. (2023) 227:236–45. doi: 10.1093/infdis/jiac366 PMC9494415 36082433

[36] Semmes EC Chen JL Goswami R Burt TD Permar SR Fouda GG. Understanding early-life adaptive immunity to guide interventions for pediatric health. Front Immunol. (2021) 11:595297. doi: 10.3389/fimmu.2020.595297 33552052 PMC7858666

[37] Joshi D Nyhoff LE Zarnitsyna VI Moreno A Manning K Linderman S. Infants and young children generate more durable antibody responses to SARS-CoV-2 infection than adults. iScience. (2023) 26:107967. doi: 10.1016/j.isci.2023.107967 37822504 PMC10562792

[38] Zhao J Yuan Q Wang H Liu W Liao X Su Y. Antibody responses to SARS-CoV-2 in patients of novel coronavirus disease 2019. SSRN Electron J. (2020) 71:2027–34. doi: 10.1093/cid/ciaa344 PMC7184337 32221519

[39] Feng W Xiang Y Wu L Chen Z Li Q Chen J. Nucleocapsid protein of SARS-CoV-2 is a potential target for developing new generation of vaccine. J Clin Lab Anal. (2022) 36:e24479. doi: 10.1002/jcla.24479 35527696 PMC9169192

[40] Goldman AS Chheda S Garofalo R. Evolution of immunologic functions of the mammary gland and the postnatal development of immunity. Pediatr Res [Internet]. (1998) 43:155–62. doi: 10.1203/00006450-199802000-00001 9475278

[41] Mulleners SJ Juncker HG Ruhé EJM Korosi A van Goudoever JB van Gils MJ. Comparing the SARS-CoV-2-specific antibody response in human milk after homologous and heterologous booster vaccinations. Commun Biol. (2023) 6:1–7. doi: 10.1038/s42003-023-04455-4 36697496 PMC9875178

[42] Richard RM Maziashvili G Tran M Ramos I Laxman AS Didbaridze N. Breast milk conferred immunity to infants against COVID-19. Cureus. (2023) 15:e42075. doi: 10.7759/cureus.42075 37602015 PMC10434728

[43] Delpisheh A Brabin L Attia E Brabin BJ. Pregnancy late in life: a hospital-based study of birth outcomes. J Womens Health (Larchmt). (2008) 17:965–70. doi: 10.1089/jwh.2007.0511 PMC3000925 18554095

[44] Zhang C Hediger ML Albert PS Grewal J Sciscione A Grobman WA. Association of maternal obesity with longitudinal ultrasonographic measures of fetal growth: Findings from the NICHD fetal growth studies-singletons. JAMA Pediatr. (2018) 172:24–31. doi: 10.1001/jamapediatrics.2017.3785 29131898 PMC5808867

[45] Kyathanahalli C Snedden M Hirsch E. Is human labor at term an inflammatory condition?†. Biol Reprod. (2023) 108:23–40. doi: 10.1093/biolre/ioac182 36173900 PMC10060716

[46] Gomez-Lopez N StLouis D Lehr MA Sanchez-Rodriguez EN Arenas-Hernandez M. Immune cells in term and preterm labor. Cell Mol Immunol. (2014) 11:571–81. doi: 10.1038/cmi.2014.46 PMC4220837 24954221

[47] Montgomery KS. Apgar scores: Examining the long-term significance. J Perinat Educ. (2000) 9:5–9. doi: 10.1624/105812400X87716 17273212 PMC1595023

[48] Razaz N Cnattingius S Persson M Tedroff K Lisonkova S Joseph KS. One-minute and five-minute Apgar scores and child developmental health at 5 years of age: a population-based cohort study in British Columbia, Canada. BMJ Open. (2019) 9:e027655. doi: 10.1136/bmjopen-2018-027655 PMC6528022 31072859

[49] Razaz N Cnattingius S Joseph KS. Association between Apgar scores of 7 to 9 and neonatal mortality and morbidity: population based cohort study of term infants in Sweden. BMJ. (2019) 365:l1656. doi: 10.1136/bmj.l1656 31064770 PMC6503461

[50] Marwah M Shokr H Demitry A Wang K Ahmad S Marwah S. SARS-2 COVID-19-induced immunity response, a new prognostic marker for the pregnant population correlates inversely with neonatal Apgar score. Infection [Internet]. (2022) 50:1121–9. doi: 10.1007/s15010-022-01773-3 PMC8897759 35247163

[51] Straube S Voigt M Jorch G Hallier E Briese V Borchardt U. Investigation of the association of Apgar score with maternal socio-economic and biological factors: an analysis of German perinatal statistics. Arch Gynecol Obstet. (2010) 282:135–41. doi: 10.1007/s00404-009-1217-7 PMC2896624 19714345

[52] Yang HS Costa V Racine-Brzostek SE Acker KP Yee J Chen Z. Association of age with SARS-CoV-2 antibody response. JAMA Netw Open. (2021) 4:e214302. doi: 10.1001/jamanetworkopen.2021.4302 33749770 PMC7985726

[53] Amanat F Thapa M Lei T Ahmed SMS Adelsberg DC Carreño JM. SARS-CoV-2 mRNA vaccination induces functionally diverse antibodies to NTD, RBD, and S2. Cell. (2021) 184:3936–3948.e10. doi: 10.1016/j.cell.2021.06.005 34192529 PMC8185186

[54] Nir O Schwartz A Toussia-Cohen S Leibovitch L Strauss T Asraf K. Maternal-neonatal transfer of SARS-CoV-2 immunoglobulin G antibodies among parturient women treated with BNT162b2 messenger RNA vaccine during pregnancy. Am J Obstet Gynecol MFM. (2022) 4:100492. doi: 10.1016/j.ajogmf.2021.100492 34547533 PMC8451978

[55] Kugelman N Nahshon C Shaked-Mishan P Cohen N Sher ML Gruber M. Maternal and neonatal SARS-CoV-2 immunoglobulin G antibody levels at delivery after receipt of the BNT162b2 messenger RNA COVID-19 vaccine during the second trimester of pregnancy. JAMA Pediatr. (2022) 176:290–5. doi: 10.1001/jamapediatrics.2021.5683 PMC8693209 34932066

[56] Prabhu M Yang YJ Johnston CD Murphy EA Ketas TJ Diaz-Tapia R. Longitudinal antibody response kinetics following SARS-CoV-2 messenger RNA vaccination in pregnant and nonpregnant persons. Am J Obstet Gynecol MFM. (2023) 5:100796. doi: 10.1016/j.ajogmf.2022.100796 36334723 PMC9626404

[57] Kritikos A Gabellon S Pagani JL Monti M Bochud PY Manuel O. Anti-SARS-CoV-2 titers predict the severity of COVID-19. Viruses. (2022) 14:1089. doi: 10.3390/v14051089 PMC9143418 35632830

[58] Sette A Crotty S. Adaptive immunity to SARS-Cov-2 and COVID-19. Cell. (2021) 184:861–80. doi: 10.1016/j.cell.2021.01.007 PMC7803150 33497610

