# Supplementary material for: SARS-CoV-2 anti-RBD and anti-N protein responses are differentially regulated between mother-child pairs: insight from a national study cohort at the Faroe Islands
Source: Front Immunol. 2024 Jul 3;15:1418678. doi: 10.3389/fimmu.2024.1418678 (PMC11251900; doi:10.3389/fimmu.2024.1418678)

Supplementary Tables

Table S1. Statistics regarding breastfeeding and the SARS-CoV-2 Ig response in serum from children 12 month after birth

	RBD Ig levels		N protein Ig levels	
	Mean rank diff.	P-value	Mean rank diff.	P-value
**Kruskal-Wallis ANOVA**				
Differences between groups (duration of breastfeeding)		0.543		0.303
**Kruskal-Wallis with Dunn's multiple comparisons**				
Never breastfed vs. Breastfed <26 weeks	19.72	0.963	27.69	0.491
Never breastfed vs. Breastfed >26 weeks	20.90	0.808	29.24	0.367
Breastfed <26 weeks vs Breastfed >26 weeks	1.180	>0.999	1.557	>0.999
**Mann Whitney test**				
No breastfeeding vs Still breastfeeding		0.096		0.788

Table S2. Linear regression models investigating associations between maternal parameters and the SARS-CoV-2 Ig response in children 12 month after birth

	Neonatal serum at 12-month sampling[a]			
	RBD Ig levels		N protein Ig levels	
	EST	p-value	EST	p-value
**Vaccination status**	0.0594	0.3193	0.0926	0.3458
1 dose	0.0549	0.5510	0.0745	0.6293
2 doses	0.0761	0.2011	0.1003	0.3145
3 doses	-0.0054	0.9344	0.0659	0.5491
**Infection status**	-0.0230	0.6310	-0.0230	0.6310
**Breastfeeding** (intercept: never received breastmilk)				
Breastfed for less than 26 weeks	-0.0006	0.9870	-0.0144	0.8040
Breastfed for more than 26 weeks	0.0187	0.5850	-0.0561	0.3200
**Still breastfeeding**	0.0170	0.5610	-0.0119	0.8090

[a]unadjusted using linear regression

Supplementary Figures

Figure S1. A Population-based Prospective Cohort from the Faroe Islands. A study overview (a). A simplified overview of key maternal characteristics at baseline (b). Created with BioRender.com

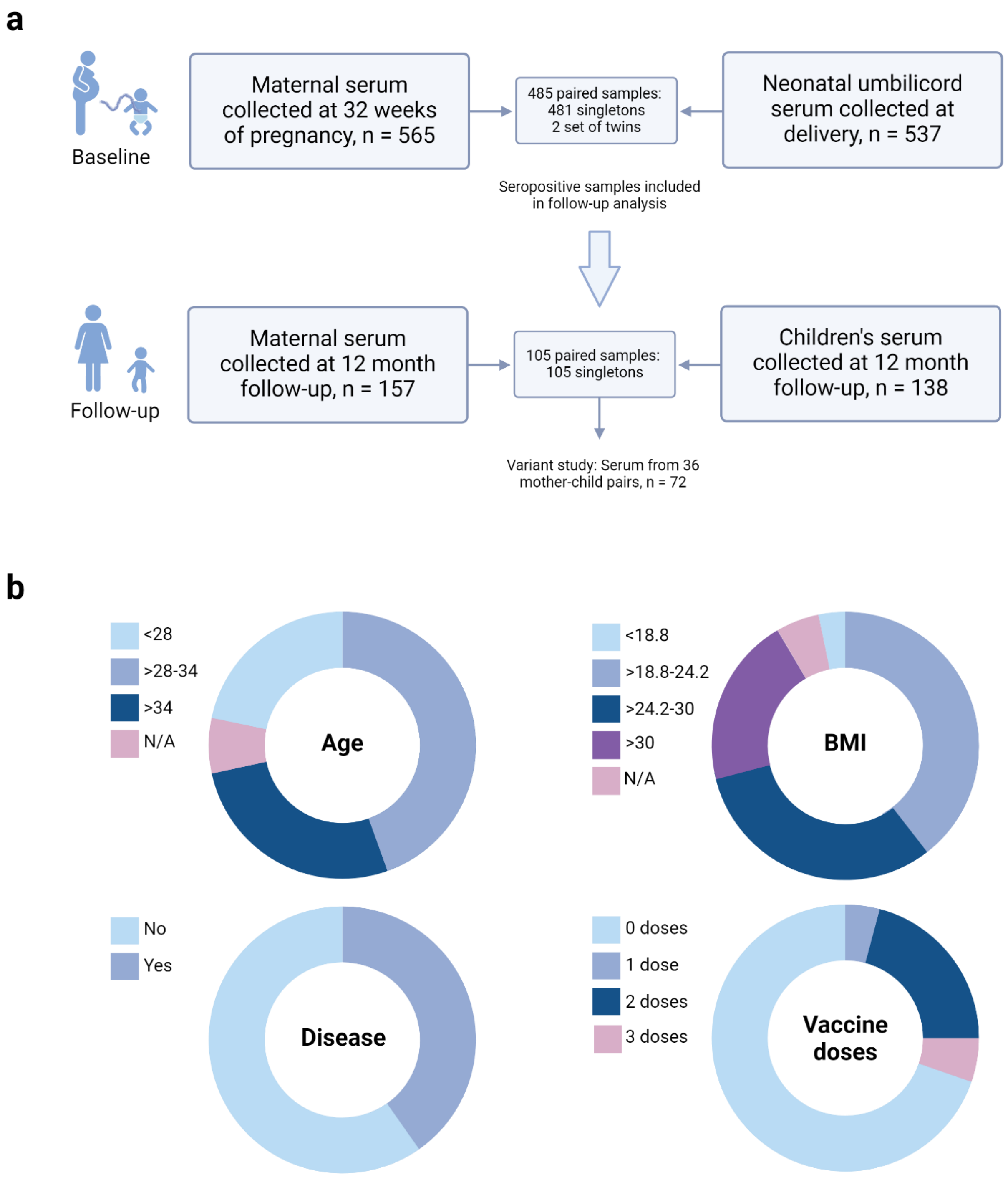

Figure S2. Re-evaluation of assay positivity threshold. ROC curve analysis was performed to study the performance of RBD and N protein-specific S-ELISA setups. The evaluation utilized a dataset comprising 100 positive samples, characterized as women with PCR-confirmed SARS-CoV-2 infection, and 100 negative controls, who were neither infected nor vaccinated.

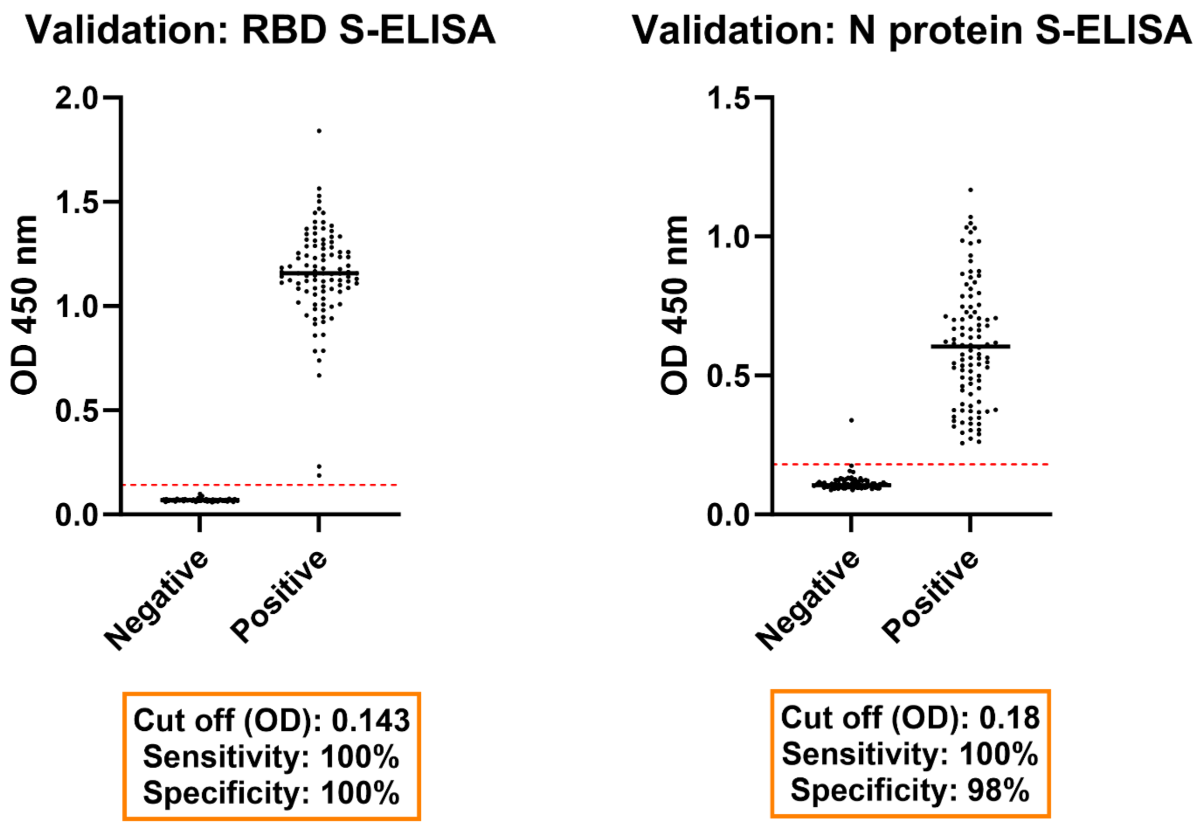

Figure S3. Distribution of SARS-CoV-2 IgG, IgM, and IgA against RBD at baseline. Levels of RBD-specific IgG, IgM and IgA in maternal serum at 32 weeks of pregnancy (a-c) and neonatal cord serum right after delivery (d-f). Green and yellow colors represent not vaccinated and vaccinated, respectively. Data reported as median and interquartile range (box), whiskers represent 1.5 times the IQR. Data are represented in log10(AU/ml) and horizontal line represents the threshold for assay positivity. P-values < 0.05 was considered statistically significant by Mann-Whitney test.

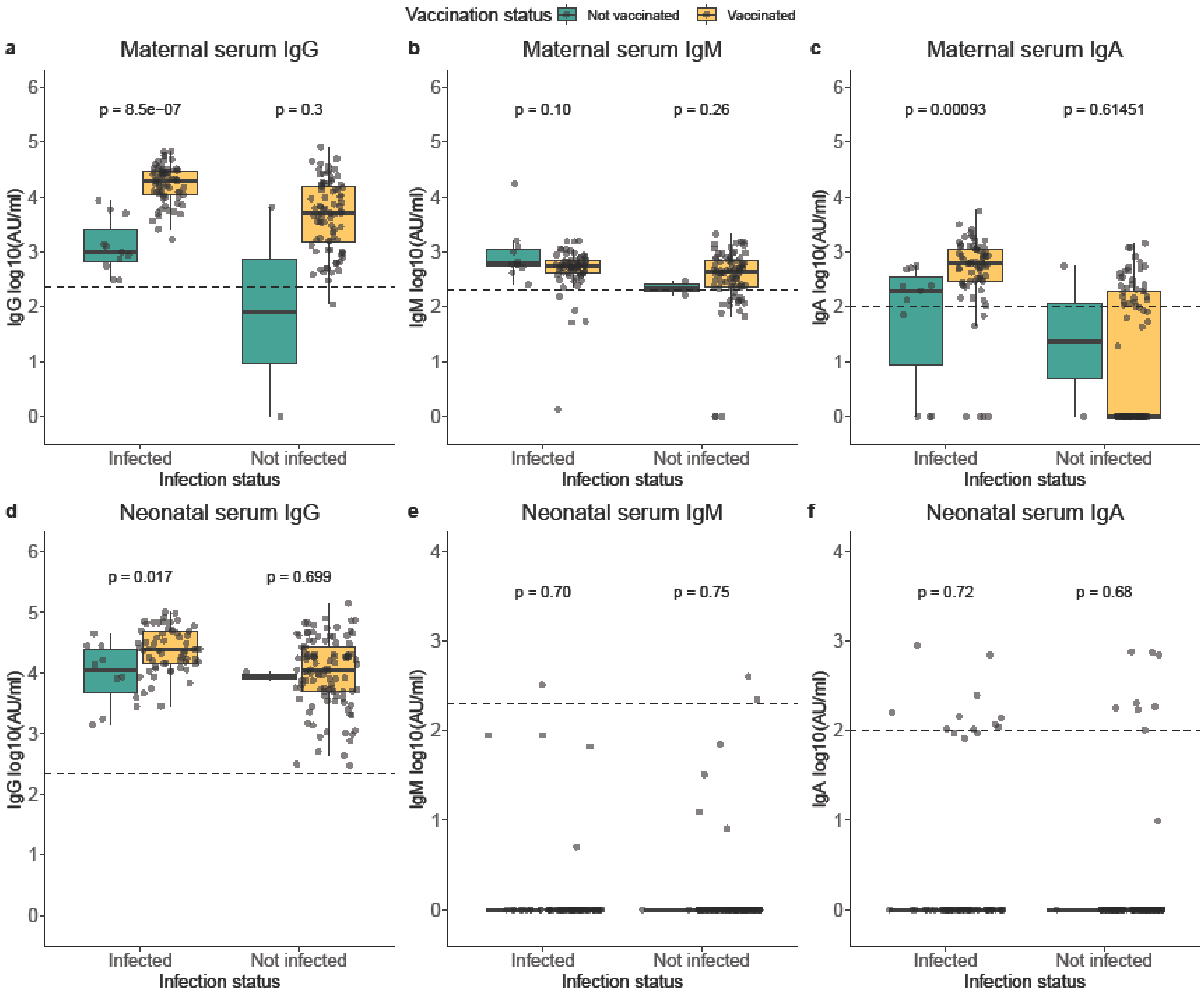

Figure S4. Distribution of SARS-CoV-2 IgG, IgM, and IgA against RBD at 12-month follow-up. Levels of RBD-specific IgG, IgM and IgA in maternal serum (a-c) and children's serum (d-f), collected 12 months after delivery. Green and yellow colors represent not vaccinated and vaccinated, respectively. Data are represented in log10(AU/ml) and horizontal line represents the threshold for assay positivity. Data reported as median and interquartile range (box), whiskers represent 1.5 times the IQR. P-values < 0.05 was considered statistically significant by Mann-Whitney test.

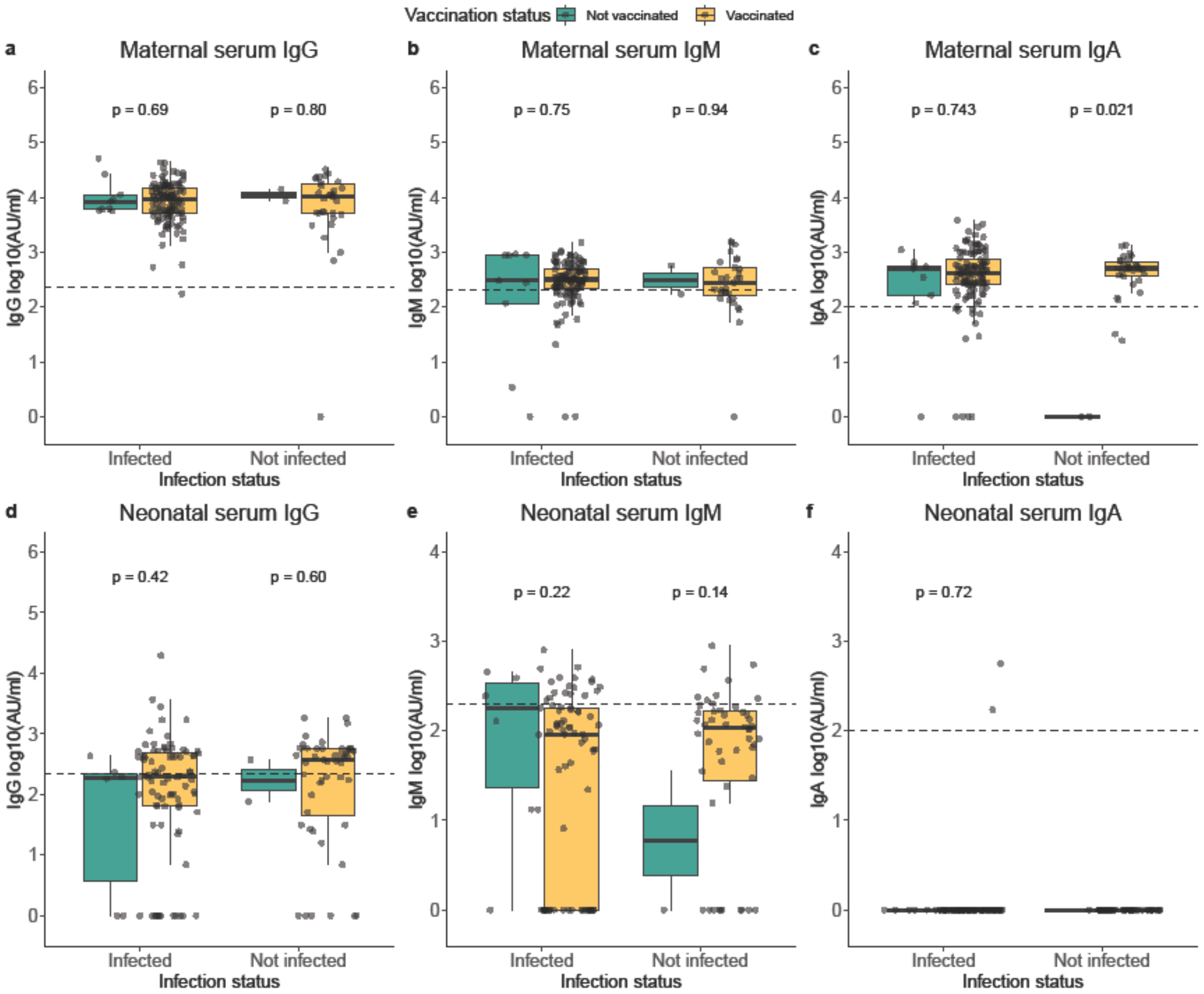

Supplement: Supplementary file 1 [file DataSheet_1.pdf]
